# Spontaneous Hemopericardium Associated With Apixaban Use With Newly Diagnosed Malignancy and Acute Kidney Injury: A Case Report

**DOI:** 10.7759/cureus.60410

**Published:** 2024-05-16

**Authors:** Awss Shalhoub, Omar M Masarweh, Nicole Brenner

**Affiliations:** 1 Internal Medicine, University of Central Florida, Kissimmee, USA

**Keywords:** malignancy, direct oral anticoagulants (doac), anticoagulation, hemopericardium, apixaban

## Abstract

Direct oral anticoagulants have simplified the use of anticoagulation for patients and clinicians. These medications now have indications for non-valvular atrial fibrillation and venous thromboembolism and carry a lower risk of bleeding than warfarin. While bleeding complications are common amongst all anticoagulants, spontaneous hemopericardium is a rarely reported side effect of direct oral anticoagulants, previously reported in patients with concomitant malignancy or kidney injury. We present a case of a patient with recently diagnosed renal malignancy and atrial fibrillation on apixaban who developed a spontaneous hemopericardium that required a pericardial window.

## Introduction

In recent years, the advent of direct oral anticoagulants (DOACs) has revolutionized the landscape of anticoagulation therapy, offering improved safety outcomes compared to traditional agents such as warfarin. DOACs, such as apixaban, are usually favored over warfarin in patients with non-valvular atrial fibrillation, venous thromboembolism, or pulmonary embolism as they do not require monitoring of therapeutic levels and they are associated with less risk of bleeding [1]. DOACs have gained popularity due to their efficacy in preventing thromboembolic events with lower risk of bleeding complications. However, adverse events may still occur, such as spontaneous hemopericardium. Herein, we present a case of a 66-year-old male with atrial fibrillation on apixaban and recently diagnosed renal malignancy who developed spontaneous hemopericardium requiring a pericardial window. This case delves into the rare occurrence of spontaneous hemopericardium associated with the use of apixaban, shedding light on an unusual and a potentially critical complication.

## Case presentation

**Figure 1:**
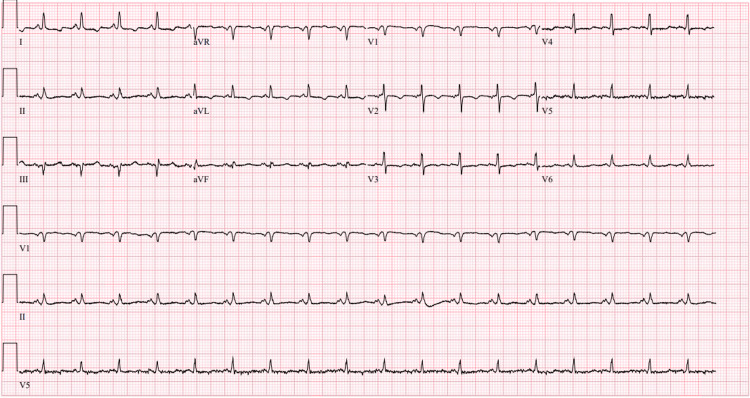
The 12 lead electrocardiogram demonstrating diffuse low voltage.

A 66-year-old man with hypertension, hyperlipidemia, Hashimoto’s thyroiditis, and atrial fibrillation chronically on apixaban 5 mg twice a day, last dose 12 hours prior to presentation, and recently diagnosed left renal cell carcinoma with planned resection one week after this presentation, presented to an outside emergency department (ED) for two to three days of right-sided abdominal pain and pleuritic chest pain. He reported experiencing worsening peri-umbilical abdominal pain that began three days prior, that migrated to the epigastric area, associated with diffuse pleuritic chest pain. He also reported lightheadedness, dizziness, decreased oral intake, nausea, fatigue, dyspnea on exertion, and orthopnea over the same period. On arrival to the outside ED, his weight was 108 kilograms, blood pressure was 140/82 mmHg, pulse rate 107 beats per minute (bpm), and oxygen saturation 96% on 2 liters by nasal cannula. Laboratory investigation revealed hemoglobin 12.3 mg/dL (reference 13.7-17.5 mg/dL), platelets 280,000/mm3 (reference 150,000-400,000/mm3), serum creatinine 1.8 mg/dL (reference 0.55-1.3 mg/dl), aspartate transaminase (AST) 126 u/L (reference 10-37 u/L), alanine transaminase (ALT) 135 u/L (reference 12-78 u/L), high-sensitivity troponin 36 ng/L (reference range <14 ng/L), brain-natriuretic peptide (BNP) 197 pg/mL (reference <100 pg/ml), international normalized ratio (INR) 2.1 (reference 0.8-1.1), and prothrombin time (PT) 17.3 seconds (reference 10.0-12.8 seconds). Electrocardiogram showed sinus tachycardia with a heart rate of 110 bpm and low voltage QRS (Figure 1). Computed tomography (CT) of the chest without contrast demonstrated a 2.6 cm pericardial effusion. CT of the abdomen and pelvis without contrast showed a 2.3 cm soft tissue density lesion in the upper pole of the left kidney. The patient was then transferred to our facility for cardiothoracic surgery evaluation. Prior to transfer, he was given 5,000 units of prothrombin complex.

**Figure 2:**
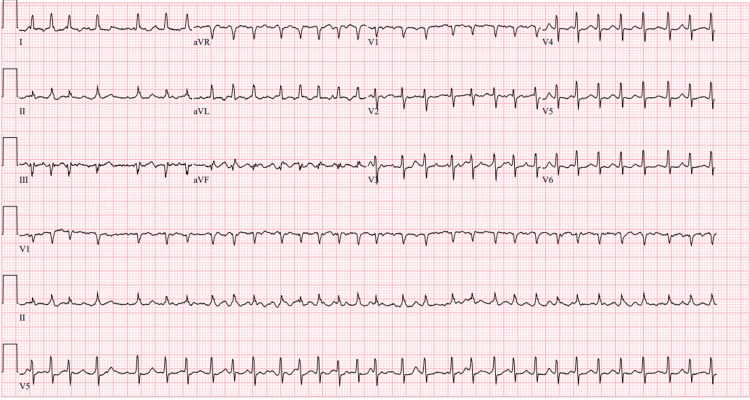
The 12 lead electrocardiogram demonstrating atrial fibrillation with rapid ventricular response with low voltage and electrical alternans.

Upon arrival to our facility, repeat findings were blood pressure 118/72 mmHg, pulse rate 104 bpm, oxygen saturation 97% on 2 liters nasal cannula, and he was transferred to the cardiovascular intensive care unit. The patient then developed intermittent atrial fibrillation with rapid ventricular response, with rates of 184 bpm that were short-lived and self-terminating. His blood pressure remained stable (Figure 2).

**Figure 3:**
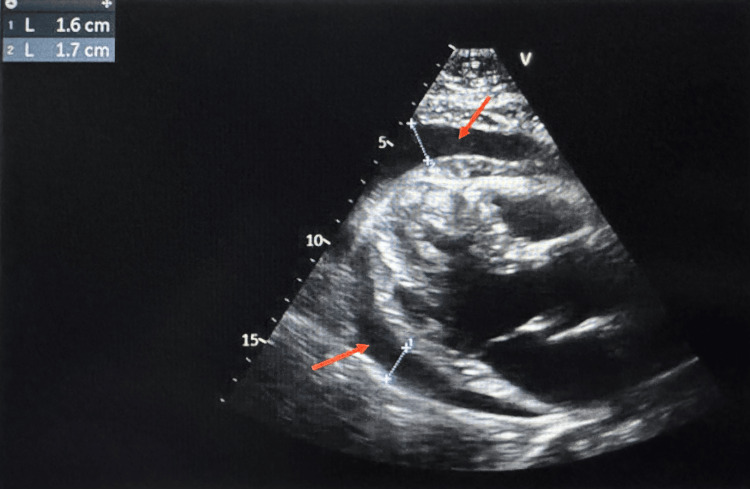
Transthoracic echocardiogram, parasternal long axis view, showing pericardial effusion (red arrows) measuring 1.7 cm (blue dotted lines).

**Figure 4:**
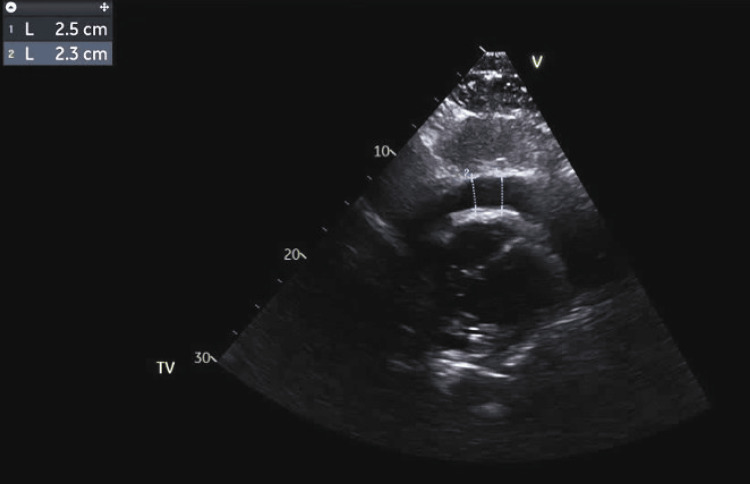
Transthoracic echocardiogram, parasternal short axis view, showing a large pericardial effusion measuring 2.5 cm (blue dotted lines).

An urgent echocardiogram showed a large pericardial effusion but no evidence of chamber collapse or tamponade physiology, with an estimated ejection fraction of 25-30% (Figures 3, 4).

Repeat laboratory investigation revealed worsening renal function with a serum creatinine of 2.4 mg/dL, hemoglobin 11.8 mg/dL, AST 3,187 u/L, ALT 2,536 u/L, and lactic acid 6.2 mmol/L (reference range < 2) indicating cardiogenic shock (Table 1).

**Table 1:** Laboratory values.

Laboratory test	On presentation prior to transfer	On admission to our facility	Reference range
Hemoglobin	12.3 mg/dL	11.8 mg/dL	13.7-17.5 mg/dL
Platelets	280,000/mm3	258,000/mm3	150,000-400,000/mm3
Serum creatinine	1.8 mg/dL	2.4 mg/dL	0.5- 1.3 mg/dL
Alanine transaminase	135 u/L	2,536 u/L	12-78 u/L
Aspartate transaminase	126 u/L	3,187 u/L	10-37 u/L
International normalized ratio	2.1	3.3	0.8-1.1
Prothrombin time	17.3 seconds	24.4 seconds	10-12.8 seconds
Lactic acid	-	6.2 mmol/L	< 2.0 mmol/L
High sensitivity troponin	36 ng/L	13 ng/L	<14 ng/L
Brain natriuretic peptide	197 pg/mL	-	<100 pg/mL

The patient subsequently underwent a subxiphoid pericardial window that drained 500 ml bloody fluid. Pericardial fluid analysis, including culture, gram stain, and cytology, was negative for malignancy and infection but was consistent with hemorrhage. He remained hospitalized for five days of observation during which time his symptoms and end-organ function significantly improved. His renal function improved, reflected by a serum creatinine of 0.75 mg/dL, liver function tests normalized, and a repeat echocardiogram demonstrated persistently reduced ejection fraction of 35% without recurrence of pericardial fluid. Auto-immune and vasculitis workup, including rheumatoid factor, anti-neutrophil antibody (ANA), cytoplasmic antineutrophilic cytoplasmic antibody (c-ANCA), perinuclear antineutrophilic cytoplasmic antibody (p-ANCA), anti-mitochondrial antibody (AMA), complement components 3 and 4 (C3, C4), Scl-70 scleroderma antibody, double-stranded DNA antibody (dsDNA Ab), anti-histone antibody, and anti-smooth muscle antibody were negative. Viral evaluation for hepatitis A, B, C, and human immunodeficiency virus (HIV) was negative. The patient was given the option of resuming his anticoagulation but declined.

## Discussion

DOACs such as dabigatran, apixaban, rivaroxaban, and edoxaban, are now indicated for non-valvular atrial fibrillation, venous thromboembolism, coronary artery disease as well as peripheral artery disease. DOACs are often favored over warfarin when indicated due to the lower risk of major bleeding, no monitoring of INR is required, and the lack of dietary restrictions. Apixaban works by inhibiting platelet activation and fibrin clot formation by direct and reversible inhibition of factor Xa [2]. An obvious common side effect is bleeding, with major and minor bleeding both reported. Precautions are often utilized to reduce the major bleeding risk and chance of supratherapeutic levels including dose reduction if serum creatinine is greater than 1.5 mg/dL and either age greater than 80 years or body weight is less than or equal to 60 kg [2].

One feared complication of apixaban use is hemopericardium. Pericardial hemorrhage or hemopericardium is a relatively rare, but potentially devastating, side effect of DOACs. It has an estimated reported incidence of 0.05%. Sheikh et al. conducted a systematic review in 2022 and identified 41 reported cases of DOAC-associated hemopericardium, with rivaroxaban the culprit in 15/41 cases, followed by dabigatran in 13/41 cases, and apixaban in 11/41 cases [3]. Since first reported in 2015, multiple other cases of hemopericardium have been reported [4]. Nasir et al. reported a case of hemopericardium in a patient with chronic lymphocytic leukemia in remission [5]. Our patient with renal cell carcinoma carries an increased cancer-associated risk of bleeding with apixaban. Cytochrome-P3A4 (CYP3A4) plays a major role in apixaban metabolism [6]. Inflammation and cancer have been shown to increase inflammatory markers that may affect CYP3A4 gene expression [7]. Another proposed mechanism may be drug-drug interaction (DDI). Ferri et al. raised concerns for possible DDI between DOACs and multiple medications including ritonavir, HIV protease inhibitors, anti-HCV therapies, chemotherapeutic agents, antibiotics, antifungals, antiplatelets, antithrombotic drugs, and antiarrhythmics partially by inhibiting CYP3A4 [8]. Prior to admission, our patient’s medications included levothyroxine, gabapentin, metoprolol succinate, and pantoprazole, which have not been suggested to be associated with DDIs with DOACs. More research is warranted to further assess the pharmacology of DDIs with DOACs [8].

Pericardial bleeding should be considered in patients on anticoagulation with symptoms such as dyspnea, tachypnea, chest pain, and physical exam findings such as pulsus paradoxes, elevated jugular venous distention, hypotension, or EKG showing electrical alternans. Bedside echocardiogram has proven to be a very useful tool that can identify pericardial fluid and a formal echocardiogram can be used to identify evidence of tamponade physiology.

Ideally, andexanet A or idarucizumab are used for the reversal of bleeding in rivaroxaban and apixaban or dabigatran, respectively [2,9]. Due to the unavailability of andexanet A at the transferring facility, our patient received prothrombin complex. There is no paucity of data to guide the resumption of DOACs after gastrointestinal or intracranial bleeds, but no current guidelines exist for other types of bleeds including hemopericardium [10]. Although monitoring of serum DOAC levels is not routinely performed, if a patient elects to resume anticoagulation after a major bleeding event, serum measurements of anti-factor Xa levels or direct thrombin inhibitor assays may be utilized to prevent or quickly identify achievement of supra-therapeutic levels. Given our patient’s elevated stroke risk and the relatively rare occurrence of hemopericardium, we offered DOACS. After a discussion of the risks, benefits, and alternatives, he declined further anticoagulant therapy.

## Conclusions

Hemopericardium is a rare, but potentially devastating. Our case highlights the potential for spontaneous hemopericardium in patients receiving direct oral anticoagulants with a known renal malignancy and reminds us to remain mindful of the major and life-threatening bleeding in anticoagulated patients with concomitant bleeding risks.
